# Intranasal dexmedetomidine vs. oral midazolam for premedication in children: a systematic review and meta-analysis

**DOI:** 10.3389/fped.2023.1264081

**Published:** 2023-11-07

**Authors:** Guangxuan Zhang, Li Xin, Qingtang Yin

**Affiliations:** ^1^ Department of Anesthesiology, Hainan Branch, Shanghai Children’s Medical Center, School of Medicine, Shanghai Jiao Tong University, Sanya, Hainan, China; ^2^ Department of Pharmacy, Sun Yat-sen Memorial Hospital, Sun Yat-sen University, Guangzhou, China; ^3^ Department of Anesthesiology, Haimen District People’s Hospital, Nantong, China

**Keywords:** dexmedetomidine, midazolam, premedication, children, review

## Abstract

**Objective:**

To compare the effects of intranasal dexmedetomidine (Dex) and oral midazolam in the preoperative medication of children by using a method of meta-analysis.

**Methods:**

Cochrane Library, Pubmed, Embase, and Web of Science were searched from inception to July 2023. Randomized controlled trials (RCTs) of intranasal Dex vs. oral midazolam in pediatric premedication were collected. Stata 15.0 statistical software was used to analyze the collected data. Relative risk (RR) and 95% confidence interval (CI) were used as effect sizes.

**Results:**

A total of 11 studies with 824 children were included, containing 415 patients in the Dex group and 409 patients in the midazolam group. Compared with the oral midazolam group, the intranasal Dex group had a better preoperative sedation effect at parent-child separation (RR = 1.37, 95% CI: 1.14–1.64) and anesthesia induction (RR = 2.08, 95% CI: 1.03–4.22). In addition, there was no significant difference in the incidence of analgesia remedy (RR = 0.60, 95% CI: 0.36–1.00) the acceptance of anesthesia masks (RR = 0.97, 95% CI: 0.83–1.12), and incidence of adverse events between (RR = 0.25, 95% CI: 0.06–1.13, *P* = 0.072) between the intranasal Dex and oral midazolam groups.

**Conclusion:**

Compared with oral midazolam, intranasal Dex has better sedative effects of parent-child separation and anesthesia induction in pediatric premedication, but there was no difference in the incidence of anesthesia remedy, anesthesia mask acceptance, and incidence of adverse events. Therefore, compared with oral midazolam, intranasal Dex is a better choice for premedication in children.

## 1. Introduction

Preoperative anxiety, fear, and agitation during parent-child separation have a great impact on children's psychology, which is a problem that clinical anesthesiologists have been paying attention to for a long time. Preoperative medication is to achieve sedation, reduce anxiety in children, and create better conditions for anesthesia. Midazolam is a commonly used preoperative drug in children, and its sedative effect has been widely recognized (1). However, the adverse reactions of oral midazolam, such as postoperative cognitive dysfunction, agitation, respiratory depression, etc., make it not an ideal preoperative medication (2, 3). The acceptance of oral midazolam in children was only 70% (4).

Dexmedetomidine (Dex), as a highly selective *α*_2_ adrenergic receptor agonist, is an active isomer of medetomidine and an imidazole derivative with sedative effects (5). Dex acts on the spinal cord conduction pathway, having certain analgesic effects, anti-sensory injury effects, and enhancement of cardiac vagus nerve excitability, without significant respiratory depression (6, 7). This drug can significantly reduce the amount of anesthetics and opioids, alleviate hemodynamic response to tracheal intubation and surgical stimulation, reduce intraocular pressure, and reduce excitement and nausea during anesthesia recovery (8, 9). A study by Yuen et al. (10) reported that intranasal infusion of 0.5 and 1 µg Dex displayed a sedative effect 45–60 min later, with the peak of sedation appearing in 90–105 min and only a slight decrease in heart rate and blood pressure.

Midazolam is a lipophilic substance, forming a stable water-soluble salt in an acidic solution with pH < 4.0 (11). Under the condition of physiologic pH value, its lipophilic bases are released and quickly cross the blood-brain barrier (11). Therefore, the lipid solubility of midazolam is high, and the absorption is rapid after oral administration. The blood concentration reaches the peak in 0.5–1 h. Due to the large first-pass effect through the liver, its bioavailability is 50% (12). At present, there is no systematic study on the pharmacokinetics of Dex administered through the nose in children. Through nasal inhalation, the drug can be absorbed directly into the blood via the nasal mucosa, which can bypass the first-pass effect of the liver.

Although Lang et al. (13) conducted a meta-analysis on the preoperative administration of Dex vs. midazolam in children, the route of administration did not distinguish between oral and nasal drops. Therefore, in this study, the method of meta-analysis was utilized to investigate the clinical effect of intranasal Dex compared with oral midazolam as preoperative medication in children, in order to provide evidence-based medical evidence for preoperative medication in children.

## 2. Methods

We conducted this meta-analysis following the principles of the Preferred Reporting Items for Systematic Reviews and Meta-Analyses (PRISMA) (14).

### 2.1. Literature retrieval

Searches were conducted in Cochrane Library, Pubmed, Embase, and Web of Science from inception to July 2023. Clinical randomized controlled trials (RCTs) of intranasal Dex vs. oral midazolam were collected in pediatric preoperative medication. The search strategy was as follows: (“children” OR “child” OR “infant” OR “pediatrics” OR “adolescent”) AND “midazolam” AND (“dexmedetomidine” OR “Dex”) AND (“randomized controlled trial” OR “RCT”). The language was limited to English. Each database was retrieved independently by two researchers and then checked. If there were any disagreements, they were resolved through discussion.

### 2.2. Document selection criteria

#### 2.2.1. Inclusion criteria

(1) Study design: RCTs. (2) Study subjects: Children 1–18 years old, no allergic history to study drugs, no organ dysfunction, no arrhythmia, no congenital diseases, and no underlying lesions. (3) Intervention measures: The experimental group received intranasal Dex before operation, and the control group received oral midazolam. (4) Outcome indicators: sedation effect during parent-child separation, sedation effect during anesthesia induction, postoperative analgesia remedy, acceptance of anesthesia mask, and incidence of adverse events.

#### 2.2.2. Exclusion criteria

(1) No valid data provided. (2) Letters, reviews, and animal studies. (3) Research not published in English.

### 2.3. Literature quality evaluation and data extraction

Quality evaluation (ZGX, and XL) and data extraction (ZGX, and XL) were performed independently by two researchers. The test was confirmed to meet the inclusion criteria. The characteristics and results of the test were recorded independently, and finally cross-checked. Any differences were resolved by discussion. If the clinical trial literature was not complete, the study would be excluded. The data of all the included literature were extracted and evaluated for their legal quality. The records were screened strictly according to the inclusion criteria and further evaluated to determine whether the study was included in this meta-analysis. The quality of the included literature was assessed using the Cochrane risk bias assessment tool (15). The evaluation included the following items: whether there was random assignment; whether the assignment scheme was hidden; whether the blind method was implemented; whether the outcome data was complete; whether the study results were selectively reported; and whether there were other possible risks of bias. The results of the evaluation were presented as unclear, low, and high risk of bias.

Data were extracted from the included literature as follows: first author, year, age, weight, sample size, country, intervention in experimental and control groups, American Society of Anesthesiologists (ASA) status, type of surgery, and outcome measures.

### 2.4. Statistical analysis

Stata 15.0 software was used for statistical analysis. All five outcome indicators were dichotomous variables, so the effect size was expressed as relative risk (RR) and 95% confidence interval (CI). Heterogeneity across studies was assessed using *I*^2^ statistics and the Chi-square test. If heterogeneity across studies was not significant (*I*^2^ < 50%, and *P* > 0.05), the fixed-effects model (FEM) was adopted for meta-analysis. If heterogeneity existed (*I*^2 ^≥ 50%, or *P* ≤ 0.05), a random-effects model (REM) was selected. Egger's Test was conducted to detect publication bias. In addition, the robustness of the obtained results was verified by sensitivity analysis.

## 3. Results

### 3.1. Literature search results and features

**Figure 1:**
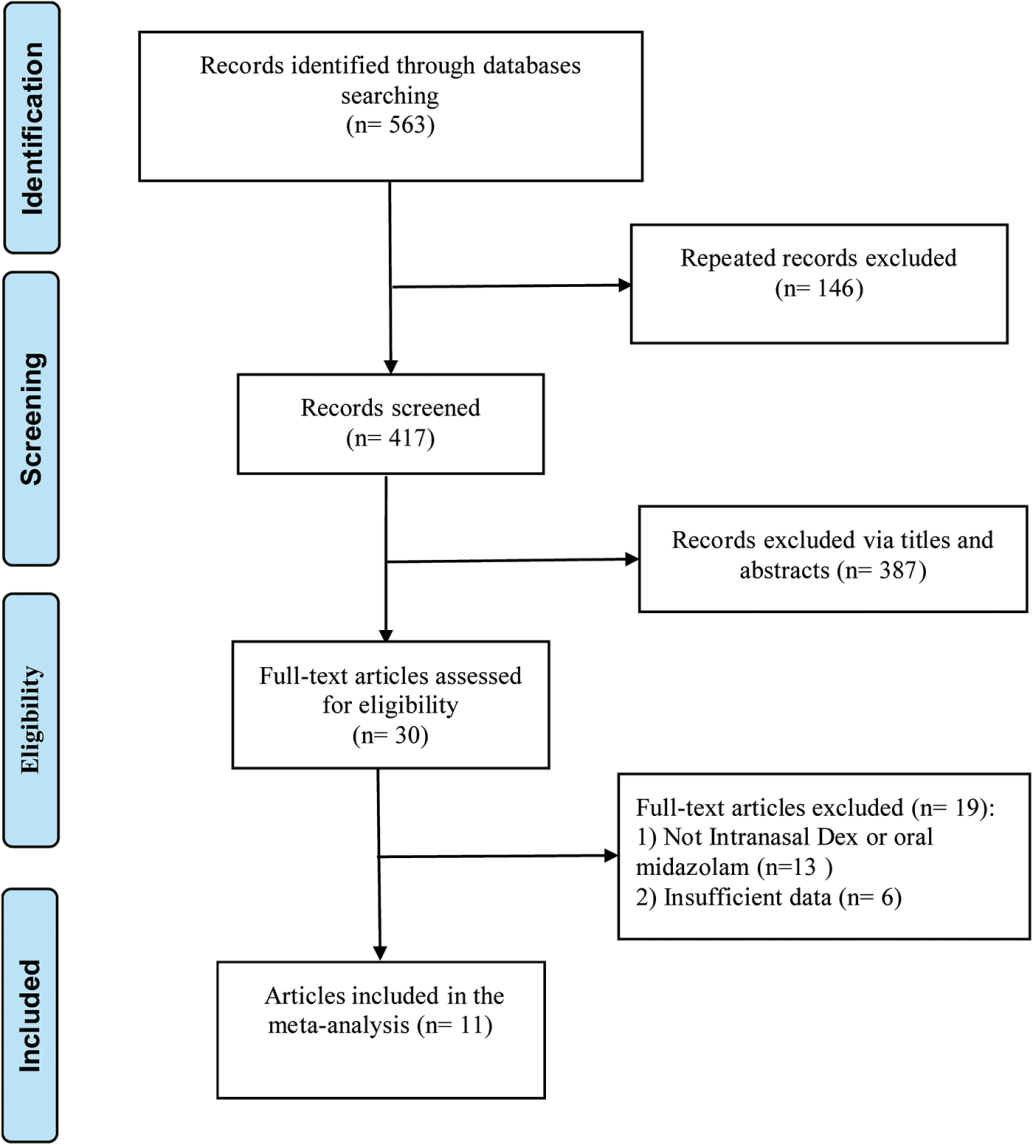
Document screening flow diagram.

**Figure 2:**
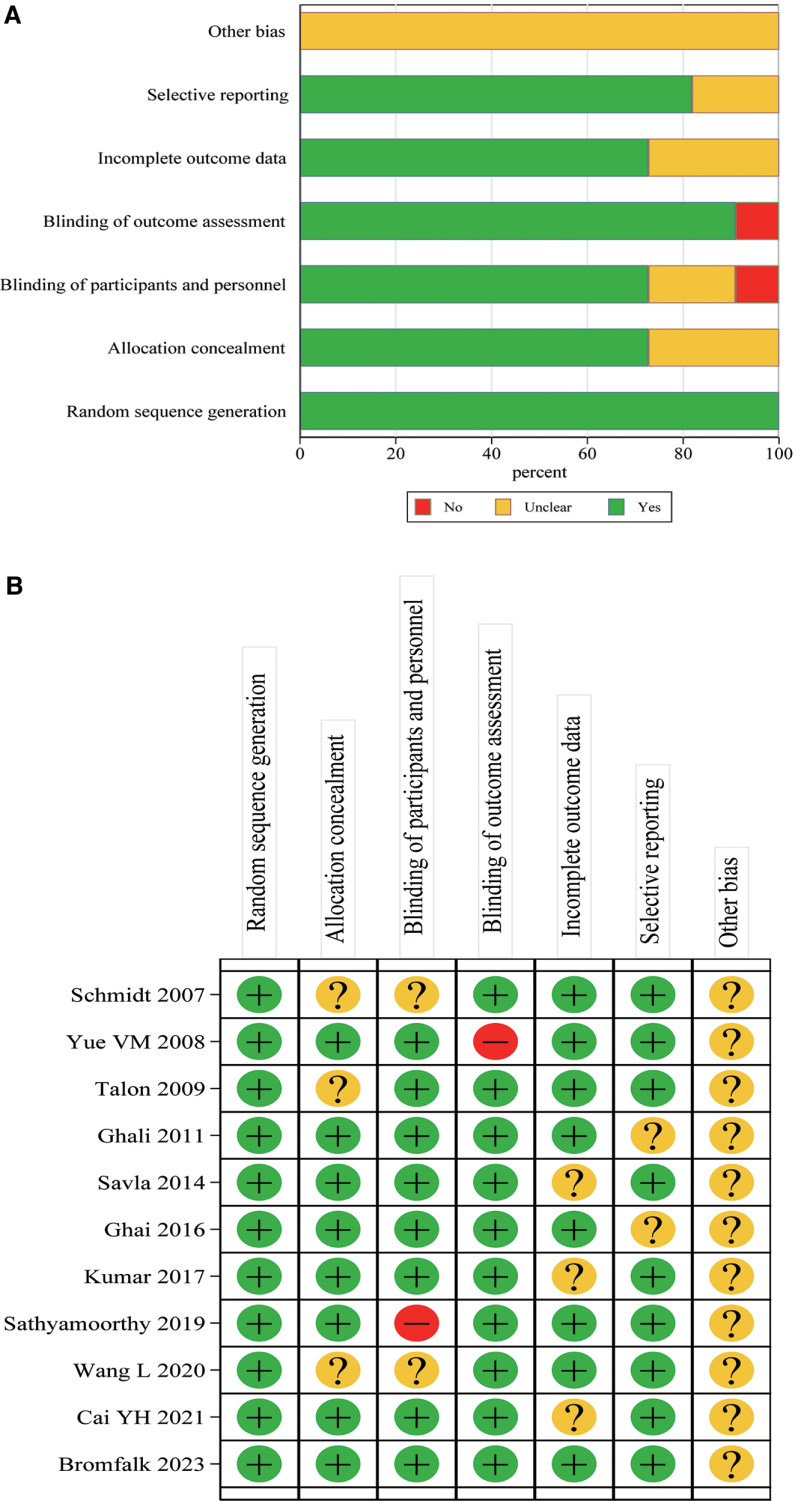
Literature quality evaluation. (**A**) Risk of bias graph; (**B**) risk of bias summary.

A total of 11 articles (16–26) were entered into this meta-analysis (Figure 1), involving 415 cases in the intranasal Dex group and 369 cases in the oral midazolam group. The basic characteristics of the included studies are shown in Table 1. The publication time of the included literature was concentrated between 2007 and 2023 of the 11 included papers, three were from India, three from China, two from the USA, and the remaining three were from Brazil, Sultanate of Oman, and Sweden. The study subjects were all younger than 18 years old. The pediatric surgery types covered elective surgical procedures, computed tomography (CT) imaging, dental procedures, elective ear, nose, and throat surgery, etc. Besides, intranasal Dex was given at a dose of 0.5–2.5 μg/kg, while oral midazolam was given at 0.5 mg/kg. The risk assessment of the included literature is shown in Figure 2. As shown in Figure 2, the 11 studies included were high-quality. All records had low risk of bias in random sequence generation, and 72.73% had low risk of bias in allocation concealment.

**Table 1:** Basic characteristics of the included studies.

Study	Year	Country	Study design	Ages (years; mean ± SD or meadian [range or IQR]) (Dex/midazolam)	Weight (mean ± SD or meadian [range or IQR]; Kg)	ASA status	Type of operation	Number of cases (Dex/midazolam)	Intervention measure		Outcomes
									Intranasal Dex	Oral midazolam	
Schmidt (16)	2007	Brazil	RCT	8 (8–10)/9 (8–11)	26 (23.5–33.9)/33 (28.2–37.3)	I–II	Elective ambulatory surgical procedures	20/22	1 μg/kg	0.5 mg/kg	③
Yue VM (20)	2008	China	RCT	6.8 ± 3.1 and 6.1 ± 2.7/6.4 ± 3.0	25.5 ± 11.9 and 21.6 ± 5.8/24.1 ± 8.6	I–II	Elective minor surgery	64/32	0.5 μg/kg, or 1 μg/kg	0.5 mg/kg	①②
Talon (19)	2009	USA	RCT	9.58 ± 4.37/10.72 ± 4.53	39.88 ± 16.79/35.51 ± 17.29	NR	Elective reconstructive surgery	50/50	2 μg/kg	0.5 mg/kg	①②③
Ghali (17)	2011	Sultanate of Oman	RCT	8.2 ± 1.4/8.1 ± 2.3	18.40 ± 4.74/17.9 ± 5.89	I	Elective outpatient adenotonsillectomy surgery	60/60	1 μg/kg	0.5 mg/kg	①③
Savla (24)	2014	India	RCT	3 (1–6)/4 (1–8)	12 (6–22)/14 (4–21)	I–II	Elective surgical procedure	19/15	2 μg/kg	0.5 mg/kg	④
Ghai (25)	2016	India	RCT	3.8 ± 2.1/3.1 ± 1.3	13.0 ± 3.8/12.6 ± 2.7	I–II	CT imaging	30/29	2.5 μg/kg	0.5 mg/kg	①
Kumar (18)	2017	India	RCT	6.4 ± 2.3/5.5 ± 1.7	20.2 ± 7.2/17.5 ± 6.8	I–II	Elective surgical procedures	30/30	1 μg/kg	0.5 mg/kg	①②④
Sathyamoorthy (26)	2019	USA	RCT	7 (5–18)/7 (5–18)	29 (20–146)/27 (20–77)	NR	Dental procedure	36/37	2 μg/kg	0.5 mg/kg	①
Wang L (21)	2020	China	RCT	4.56 ± 0.59/4.79 ± 0.48	15.12 ± 2.14/14.87 ± 1.56	I	Full-mouth dental rehabilitation	30/30	2 μg/kg	0.5 mg/kg	①④
Cai YH (22)	2021	China	RCT	4.3 (3.4–5.8)/5.1 (3.0–5.8)	18.0 (16.0–20.0)/19.0 (15.5–22.0)	I–II	Elective minor surgery	46/37	2 μg/kg	0.5 mg/kg	①④⑤
Bromfalk (23)	2023	Sweden	RCT	4.2 ± 1.0/4.2 ± 0.9	17.0 ± 2.1/18.3 ± 3.8	I–II	Elective ear, nose, and throat surgery	30/27	2 μg/kg	0.5 mg/kg	③⑤

ASA, American Society of Anesthesiologists; RCT, randomized controlled trial; NR, not reported; Dex, dexmedetomidine; CT, computed tomography; ①, sedative effects during parent-child separation; ②, sedative effect during anesthesia induction; ③, postoperative analgesic remedy; ④, acceptance of anesthesia masks; ⑤, incidence of adverse events; IQR, interquartile range; SD, standard deviation.

### 3.2. Main results of meta-analysis

#### 3.2.1. Heterogeneity analysis

There was significant heterogeneity in the pooled analysis of the sedative effects of parent-child separation (*I*^2 ^= 79.3%, *P* < 0.001) and anesthesia induction (*I*^2 ^= 81.7%, *P* < 0.001), so the REM was utilized for analysis. In the combined analysis of postoperative analgesic remedy (*I*^2 ^= 0.0%, *P* = 0.555), acceptance of anesthesia mask (*I*^2 ^= 0.0%, *P* = 0.828), and incidence of adverse events (*I*^2 ^= 0.0%, *P* = 0.381), no heterogeneity existed, so a FEM was used.

#### 3.2.2. Sedative effects during parent-child separation

**Figure 3:**
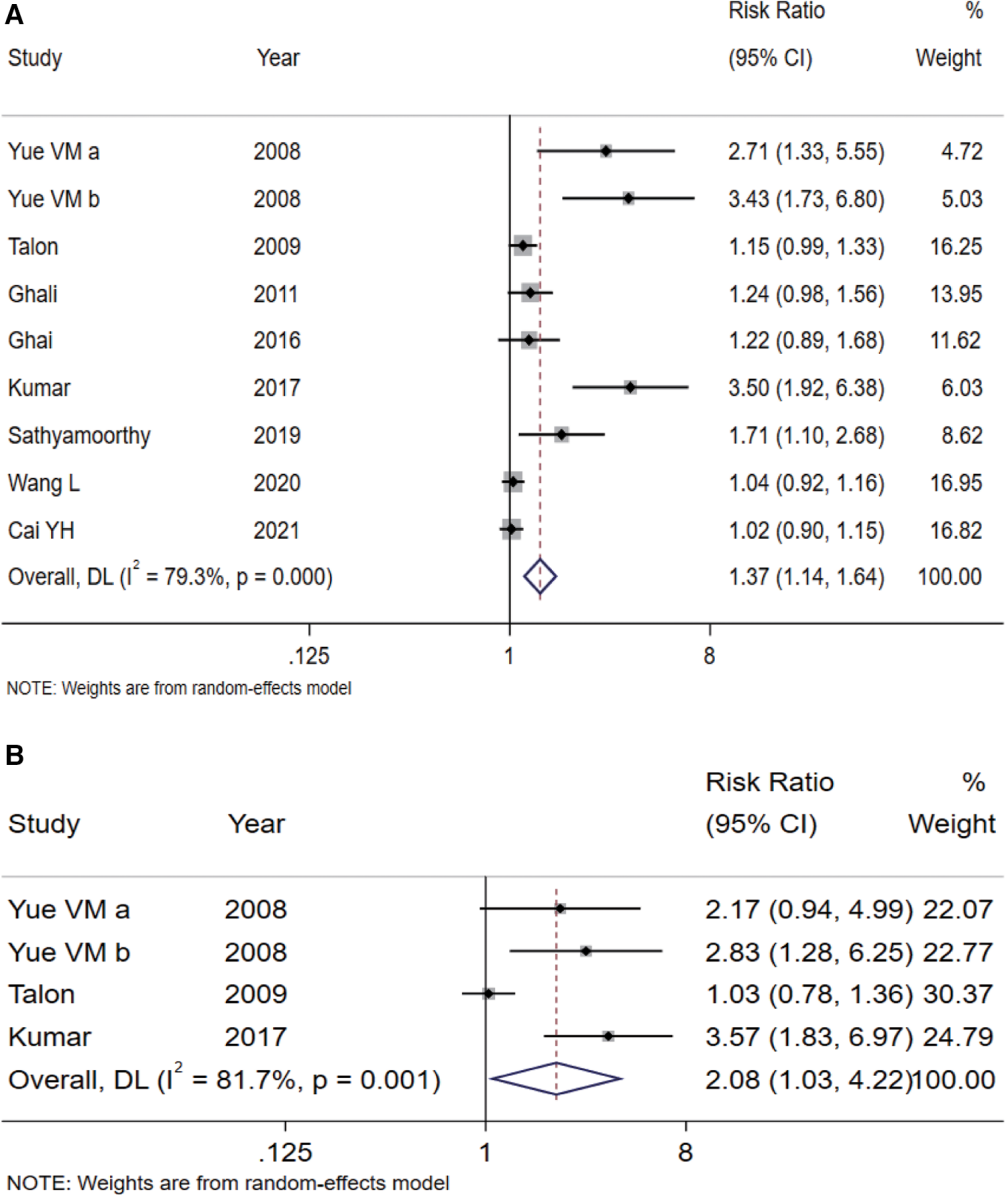
Forest plots of sedative effects during parent-child separation (**A**) and during anesthesia induction (**B**) using intranasal Dex vs. oral midazolam for premedication in children. Dex, dexmedetomidine.

A total of eight articles (including nine studies) (17–22, 25, 26) compared the effects of sedation during parent-child separation. The meta-analysis results (Figure 3A) showed that children in the intranasal Dex group had a higher percentage of parent-child separation scores (not afraid, cooperative, or asleep) and had better sedation than those in the oral midazolam group (RR = 1.37, 95% CI: 1.14–1.64, *P* = 0.001). The Egger's Test (*P* < 0.001) suggested publication bias.

#### 3.2.3. Sedative effect during anesthesia induction

A total of three articles (including four studies) (18–20) compared the effects of sedation during anesthesia induction. The pooled results (Figure 3B) indicated that compared with the oral midazolam group, the children in the intranasal Dex group had better sedation during anesthesia induction (RR = 2.08, 95% CI: 1.03–4.22, *P* = 0.043). The Egger's Test (*P* = 0.08) suggested no significant publication bias.

#### 3.2.4. Postoperative analgesic remedy

**Figure 4:**
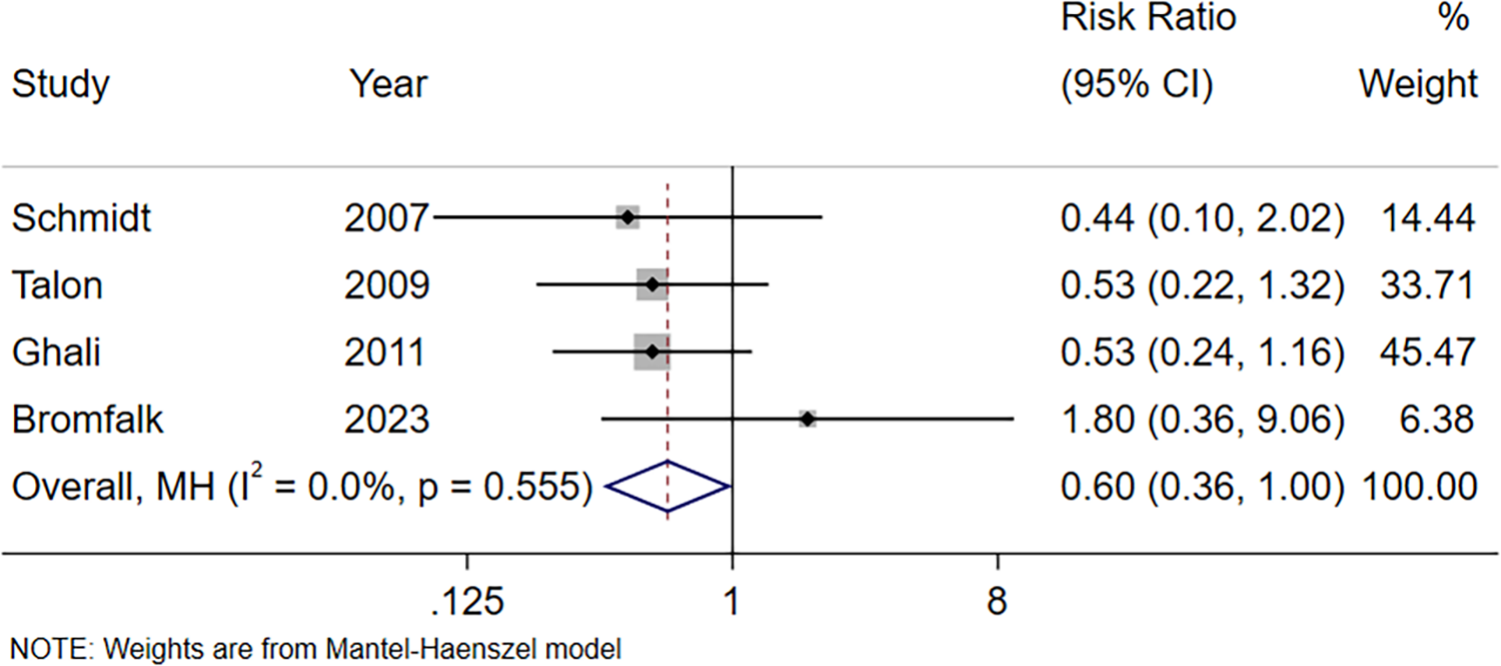
Forest plot of postoperative analgesic remedy using intranasal Dex for pediatric premedication. Dex, dexmedetomidine.

Four studies compared the incidence of postoperative analgesic remedies (16, 17, 19, 23). The meta-analysis results (Figure 4) showed that there was no difference in the incidence of postoperative analgesic remedy in the intranasal Dex group compared with the oral midazolam group (RR = 0.60, 95% CI: 0.36–1.00, *P* = 0.051). The Egger's Test (*P* = 0.441) suggested no obvious publication bias.

#### 3.2.5. Acceptance of anesthesia masks

**Figure 5:**
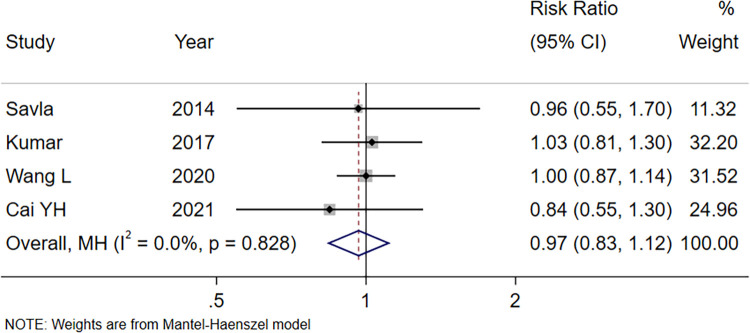
Forest plot of acceptance of anesthesia masks using intranasal Dex for pediatric premedication. Dex, dexmedetomidine.

Four studies compared the acceptance of anesthesia masks in children after preoperative medication (18, 21, 22, 24). The meta-analysis results (Figure 5) showed that there was no significant difference in the acceptance of anesthesia masks between the intranasal Dex group and the oral midazolam group (RR = 0.97, 95% CI: 0.83–1.12, *P* = 0.651). The Egger's Test (*P* = 0.404) indicated no significant publication bias.

#### 3.2.6. Incidence of adverse events

**Figure 6:**
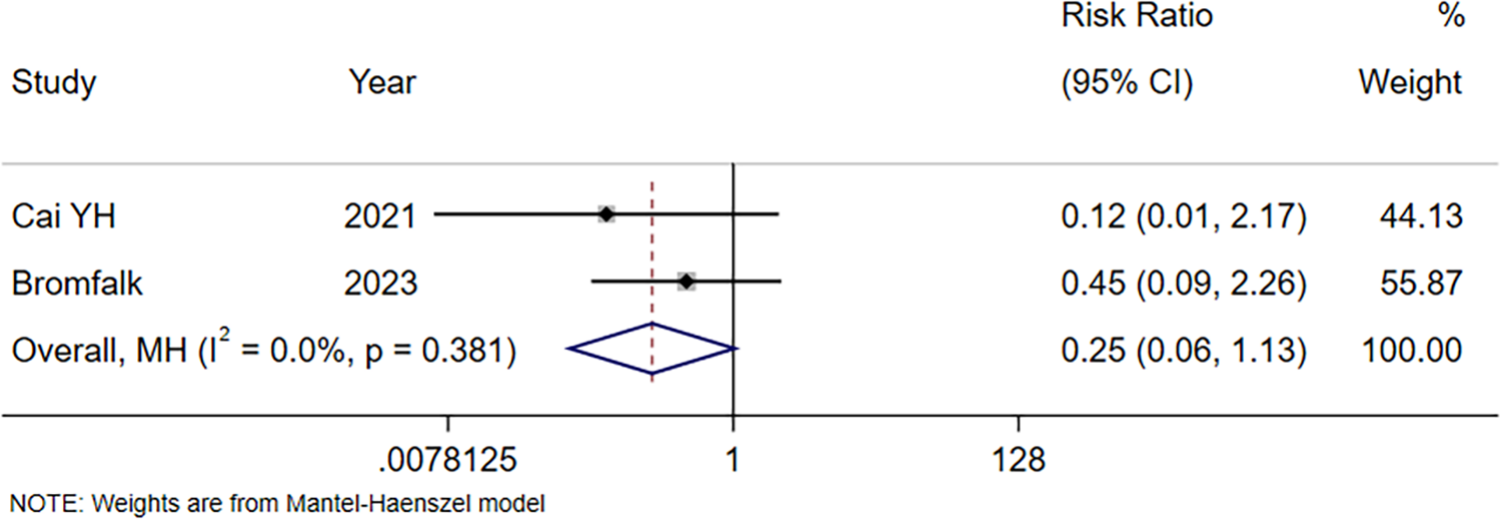
Forest plot of incidence of adverse events using intranasal Dex for pediatric premedication. Dex, dexmedetomidine.

Two records reported the incidence of adverse events (22, 23). The meta-analysis results illustrated no significant difference in the incidence of adverse events between the intranasal Dex group and the oral midazolam group (RR = 0.25, 95% CI: 0.06–1.13, *P* = 0.072; Figure 6).

### 3.3. Sensitivity analysis

**Figure 7:**
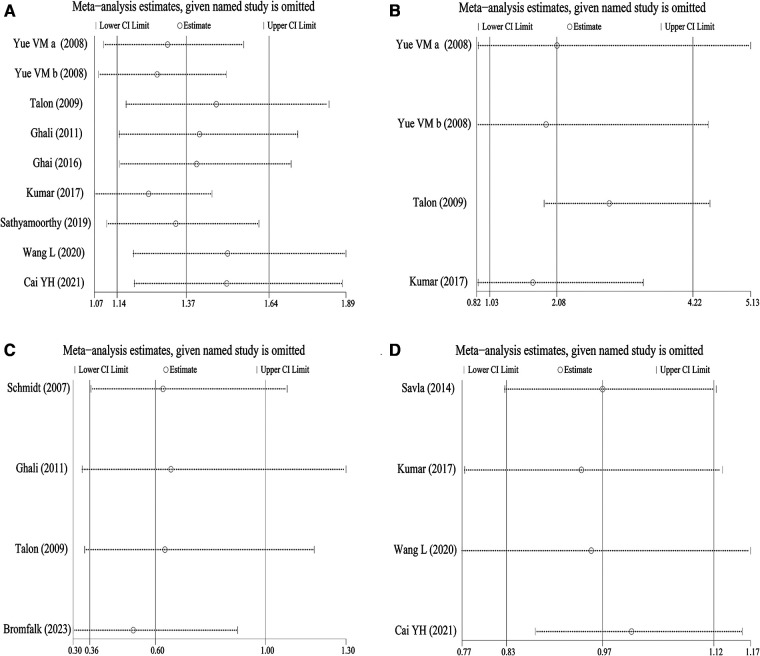
Sensitivity analysis to detect robustness of the findings. (**A**) Sedative effect during parent-child separation; (**B**) sedative effect during anesthesia induction; (**C**) postoperative analgesic remedy; (**D**) acceptance of anesthesia masks.

After each record was removed, the results of the meta-analysis were compared again with those before the removal to conduct the sensitivity analysis. The results (Figures 7A–D) showed that in the analysis of the sedation effect and acceptance of anesthesia masks during parent-child separation, there was no significant change in the results of meta-analysis after excluding any studies. However, in the analysis of the sedation effect during anesthesia induction and postoperative analgesia remedy, when 2 (18, 20) and 1 (23) articles were omitted, respectively, the conclusions were significantly changed. This suggested that the results obtained in the meta-analysis of sedation effects during anesthesia induction and postoperative analgesia remedies were not robust to some extent.

## 4. Discussion

Children with extreme anxiety and fear during anesthesia induction may experience a range of adverse clinical outcomes, such as post-awakening delirium, an increased need for analgesics, and the appearance of postoperative adverse reactions. Sleep disorders, separation anxiety, and eating problems are common manifestations of postoperative adverse reactions (27, 28). In addition, perioperative stress may also lead to poor adherence to medication. Therefore, it is essential to reduce preoperative anxiety in children to prevent long-term behavioral changes in children. Sathyamoorthy et al. (26) reported that the mean systolic blood pressure and heart rate in the intranasal Dex group were significantly lower than those in the oral midazolam group after 45 min of pediatric premedication. Bromfalk et al. (23) showed that the Ramsay Sedation Scale scores in the intranasal Dex group were significantly higher than those in the oral midazolam group after 60 min of pediatric premedication.

The results of this meta-analysis suggested that intranasal Dex had a better sedative effect on preoperative administration in children than oral midazolam. In particular, the differences in the sedation effect during parent-child separation and the sedation effect during anesthesia induction were statistically significant. However, there was no difference between oral midazolam and intranasal Dex in terms of postoperative analgesic remedy, anesthesia mask acceptance, and incidence of adverse events. Sensitivity analysis confirmed that the results of mask acceptability and sedation effect during parent-child separation were robust. It is necessary to note that conclusions about the incidence of postoperative analgesia remedies need to be treated with caution based on current results. Sensitivity analysis showed that the conclusion about postoperative analgesia remedies has significantly changed after the exclusion of one article. Therefore, more research is needed to confirm this result. The Egger's Test of mask acceptability, post-operative analgesic remedies, and sedation effects during anesthesia induction suggested no publication bias. A meta-analysis by Pasin et al. (29) showed that Dex had a better sedative effect than midazolam on the parent-child separation regardless of drug route, while there was no statistical difference between Dex and midazolam on the sedative effect of anesthesia induction. In 2020, a meta-analysis by Lang et al. (13) reported that the sedative effect of Dex was better than that of midazolam in the parent-child separation regardless of drug route, and there was no statistical difference between Dex and midazolam in the sedative effect or mask acceptance during anesthesia induction. In addition, Lang et al. (13) also showed that the incidence of postoperative analgesic remedy in the Dex group was lower than that in the midazolam group. For sedative effects during parent-child separation, the conclusions of Pasin et al. and Lang et al. studies are consistent with those of our meta-analysis. Regarding sedation during anesthesia induction, our meta-analysis showed that the nasal effect of Dex was superior to oral midazolam, which was different from Pasin et al. and Lang et al. conclusion. The reason might be that our meta-analysis limited the drug route to intranasal Dex and oral midazolam.

Different from traditional sedatives, Dex inhibits the release of norepinephrine and reduces the electrical activity of the brain by acting on the *α*_2_ adrenergic receptor of the nucleus coeruleus of the brain stem, and finally produces a good sedative and hypnotic effect through a series of cascade reactions (30). Thus, Dex produces a sedative effect similar to physiological sleep, allowing patients to quickly and easily wake up from sedation. This partly explains why children in the Dex group were quieter, more communicative, and more cooperative during parent-child separation and induction of anesthesia. This is perhaps the most obvious advantage of Dex for pediatric anesthesia over other traditional anesthetics.

Inevitably, this meta-analysis had several limitations. First, the literature included in this study was only published in English, and did not include potentially high-quality studies published in other languages, which might lead to certain publication bias. Second, there were few records included regarding the sedation effect during anesthesia induction, mask acceptability, postoperative analgesia remedy, and incidence of adverse events, and only 3, 4, 4, and 2 articles were included, respectively, which might have some influence on the robustness of the conclusions. Third, there was a certain publication bias in the analysis of sedation effects during parent-child induction. Fourth, the results of sedation effect during anesthesia induction and postoperative analgesic remedy were not robust. Fifth, there was significant heterogeneity in the analysis of sedation effects during parent-child induction and anesthesia induction. Different study populations, differences in age and severity of basic diseases, and different medication routes and dosages might have certain effects on heterogeneity. However, the limited information in the included literature limited us to further explore the sources of heterogeneity.

In conclusion, intranasal Dex is a better choice for premedication than midazolam oral administration in Children. In particular, the sedative effect of intranasal Dex was significantly better than that of oral midazolam during parent-child separation and anesthesia induction. Besides, no significant difference was observed in the incidence of anesthesia remedy, anesthesia mask acceptance, and incidence of adverse events. However, considering the limitations of this review, such as small sample size and large heterogeneity, more researches with more rigorous study design are needed to verify the findings of this meta-analysis in the future.

## Data Availability

The raw data supporting the conclusions of this article will be made available by the authors, without undue reservation.
